# ABCG2 expression is related to low 5-ALA photodynamic diagnosis (PDD) efficacy and cancer stem cell phenotype, and suppression of ABCG2 improves the efficacy of PDD

**DOI:** 10.1371/journal.pone.0216503

**Published:** 2019-05-13

**Authors:** Noriko Kawai, Yoshihiko Hirohashi, Yuma Ebihara, Takuma Saito, Aiko Murai, Takahiro Saito, Tomohide Shirosaki, Terufumi Kubo, Munehide Nakatsugawa, Takayuki Kanaseki, Tomohide Tsukahara, Toshiaki Shichinohe, Liming Li, Satoshi Hirano, Toshihiko Torigoe

**Affiliations:** 1 Department of Pathology, Sapporo Medical University School of Medicine, Sapporo, Hokkaido, Japan; 2 Department of Gastroenterological Surgery II, Hokkaido University Graduate School of Medicine, Sapporo, Hokkaido, Japan; 3 Graduate School of Photonic Science, Chitose Institute for Science and Technology, Chitose, Hokkaido, Japan; Cedars-Sinai Medical Center, UNITED STATES

## Abstract

Photodynamic diagnosis/therapy (PDD/PDT) are novel modalities for the diagnosis and treatment of cancer. The photosensitizer protoporphyrin IX is metabolized from 5-aminolevulinic acid (5-ALA) intracellularly, and PDD/PDT using 5-ALA have been approved in dermatologic malignancies and gliomas. However, the molecular mechanism that defines the efficacy of PDD/PDT is unknown. In this study, we analyzed the functions of ATP-binding cassette (ABC) transporters in PDD using 5-ALA. Most of the human gastrointestinal cancer line cells examined showed a homogenous staining pattern with 5-ALA, except for the pancreatic cancer line PANC-1, which showed heterogeneous staining. To analyze this heterogeneous staining pattern, single cell clones were established from PANC-1 cells and the expression of ABC transporters was assessed. Among the ABC transporter genes examined, *ABCG2* showed an inverse correlation with the rate of 5-ALA-positive staining. PANC-1 clone #2 cells showed the highest level of *ABCG2* expression and the lowest level of 5-ALA staining, with only a 0.6% positive rate. Knockdown of the *ABCG2* gene by small interfering RNAs increased the positive rate of 5-ALA staining in PANC-1 wild-type and clone cells. Interestingly, PANC-1 clone #2 cells showed the high sphere-forming ability and tumor-formation ability, indicating that the cells contained high numbers of cancer stem cells (CSCs). Knockdown or inhibition of *ABCG2* increased the rate of 5-ALA staining, but did not decrease sphere-forming ability. These results indicate that gastrointestinal cancer cell lines expressing high levels of ABCG2 are enriched with CSCs and show low rates of 5-ALA staining, but 5-ALA staining rates can be improved by inhibition of ABCG2.

## Introduction

Photodynamic diagnosis/therapy (PDD/PDT) are novel modalities to detect cancer cells based on the principle that a photosensitizer can accumulate specifically in cancer cells. Recently, PDD and PDT with 5-aminolevulinic acid (5-ALA) have been used for the diagnosis of glioma [1–4], and the application of PDD/PDT is expanding to various cancers. PDD is an especially useful approach for intraoperative cancer diagnosis, such as gastric cancer and pancreatic cancer, and can detect intraperitoneal lymph node metastasis or peritoneal metastasis because the presence of peritoneal dissemination or distant lymph node metastasis is critical for the surgical approach used, and the accessibility of pathological diagnosis using frozen sections is often limited.

The detection rate of PDD is essential for its successful application; however, its sensitivity is varied in various cancers and its detection rate is sometimes low and not satisfactory [5–8]. Thus, the identification of novel biomarkers for PDD and the improvement of the success rate of PDT are important. 5-ALA is transported into the cytoplasm by the amino acid transporters PEPT1 and PEPT2, and the intermediate coproporphyrinogen III is synthesized. Coproporphyrinogen III is transported from the cytoplasm to the mitochondria by the ATP-binding cassette (ABC) transporter ABCB6. In the mitochondria, the photosensitizer protoporphyrin IX (PpIX) is synthesized from coproporphyrinogen III. PpIX is exported by ABCG2 from the mitochondria to the cytoplasm and from the cytoplasm to the extracellular space [9]. Thus, several molecules are involved in the accumulation of PpIX, and several approaches have been tried to improve the efficacy of 5-ALA PDD/PDT [10, 11]. Recent studies have demonstrated that 5-ALA PDD is useful for the detection of lymph node metastasis and peritoneal dissemination, indicating that this approach can be a powerful tool during surgical treatment [6, 12].

Cancer stem cells (CSCs) are defined as small subpopulation of cancer cells that are endowed with high tumorigenicity, capacity for self-renewal, and differentiation ability [13], and CSCs are resistant to chemotherapy and radiotherapy due to several molecular mechanisms, namely, high expression of anti-apoptosis proteins, dormant state, and high expression of transporters [14, 15]. Therefore, an effective treatment for CSCs is essential to improve current cancer therapy.

In this study, we investigated the efficacy of 5-ALA PDD using gastrointestinal cancer cell lines. We found that the pancreatic cancer cell line PANC-1 showed lower PpIX accumulation than the other cell lines examined. Analysis at clone level revealed that high ABCG2 expression was responsible for the lower accumulation of PpIX. Furthermore, ABCG2-high clone cells were enriched with CSCs, and inhibition of ABCG2 improved 5-ALA PDD. Thus, ABCG2 might be a novel target to improve the detection and therapeutic efficacy of 5-ALA PDD/PDT for CSCs.

## Materials and methods

### Ethics statement

Mice were maintained and experimented on in accordance with the guidelines of and after approval by the Committee of Sapporo Medical University School of Medicine, Animal Experimentation Center under permit number (08–006). Any animal found unhealthy or sick was promptly euthanized.

### Cell lines and culture methods

The human pancreas cancer cell lines CFPAC and PANC-1 (American Type Culture Collection, Rockville, MD) were cultured in Dulbecco’s modified Eagle’s medium (DMEM; Sigma-Aldrich, St. Louis, MO) supplemented with 10% fetal bovine serum (FBS; Sigma-Aldrich) and 5% penicillin-streptomycin (5 mg/mL penicillin, 5 mg/mL streptomycin; Thermo Fisher Scientific, Waltham, MA). The gastric cancer cell line MKN45 (Japanese Collection of Research Bioresources Cell Bank, Osaka, Japan), esophagus cancer cell lines TE4 and TE9 (Japanese Cell Resource Center for Biomedical Research, Sendai, Japan), and pancreas cancer cell line PK9 (Cell Resource Center for Biomedical Research, Institute of Development, Aging and Cancer, Tohoku University, Sendai, Japan) were cultured in RPMI-1640 medium (Sigma-Aldrich) supplemented with 10% FBS and 5% penicillin-streptomycin.

### 5-ALA staining and fluorescence-activated cell sorting analysis

The cell lines were suspended at 1.0 × 10^6^ cells/mL in DMEM supplemented with 5% FBS. 5-ALA (Novel Pharma, Tokyo, Japan) was added to the suspended cells at a final concentration of 150 μg/mL and incubated for 4 h at 37°C. The cells were washed with 1× phosphate-buffered saline (PBS) twice and analyzed using a fluorescence-activated cell sorting (FACS) Aria II (BD Biosciences, San Jose, CA). PpIX fluorescence was detected at excitation and emission wavelengths of 488 nm laser with a 695/40 nm band-pass filter. 5-ALA non-treated cells were used as negative controls.

Percent mean fluorescent intensity (%MFI) increase was calculated by following formula: %MFI increase = (MFI of 5-ALA treated sample)*100/(MFI of 5-ALA non-treated sample)

### Establishment of PANC-1 clone cells

PANC-1 cells were suspended at 1.0 × 10^6^ cells/mL in DMEM supplemented with 5% FBS, stained with 5-ALA for 4 h at 37°C, and sorted into single cells into 96-well plates using a FACS Aria II. After culture for several weeks, seven clone cell lines were established and used for further analysis.

### RT-PCR and quantitative RT-PCR

Total RNA samples were extracted using an RNeasy Mini Kit (QIAGEN, Germantown, MD), and cDNA samples were synthesized with 1 μg total RNA using Superscript III Reverse Transcriptase (Thermo Fisher Scientific) in accordance with the manufacturer’s protocol. PCR was performed using Taq DNA polymerase (QIAGEN) and the following thermal cycling conditions: initial denaturation for 2 min at 94°C, followed by 35 cycles of denaturation for 15 s at 94°C, annealing for 30 s at 55°C, and elongation for 30 s at 72°C, and a final elongation step for 5 min at 72°C. The primer pairs used for RT-PCR analysis are in S1 Table and previous reports [16, 17].

Quantitative RT-PCR (qRT-PCR) was performed using an ABI PRISM 7000 Sequence Detection System (Thermo Fisher Scientific) in accordance with the manufacturer’s protocol. The *ABCG2* probe was designed by the manufacturer (TaqMan Gene expression assays; Thermo Fisher Scientific), and thermal cycling was performed under the following conditions: 45 cycles of 95°C for 15 s and 60°C for 1 min. Each experiment was performed in triplicate, and *GAPDH* was used for internal normalization.

### Small interfering RNA transfection and treatment with an ABCG2 inhibitor

Pre-designed small interfering RNAs (siRNAs) targeting *ABCG2* were purchased (Silencer Select siRNA; s18056, s18057, s18058) from Thermo Fisher Scientific, and siRNA transfection was performed using the Lipofectamine RNAiMAX reagent (Thermo Fisher Scientific) in accordance with the manufacturer’s protocol. The cells were seeded in 6-well plates at 3.0 × 10^5^ cells/well in 2 mL DMEM supplemented with 10% FBS and 5% penicillin-streptomycin. Negative control siRNA (Silencer Select) was used as a negative control. Cells transfected with 20 nM siRNAs were harvested at 2 days after transfection and used for some experiments.

Ko143 (Santa Cruz Biotechnology, Dallas, TX), an ABCG2 inhibitor, was used at a concentration of 10 or 20 μM for 24 h.

### Side population assay, sphere-forming assay and Mouse xenograft assay

Side population (SP) and sphere-forming assays were performed as described previously [18, 19]. Briefly, PANC-1 wild-type (W/T) cells, clone #2 cells, and clone #4 cells were stained with Hoechst 33342 (Lonza, Basel, Switzerland) at a final concentration of 2.5 μg/mL for 60 min at 37°C. After washing with PBS, the cells were analyzed using a FACS Aria II. Verapamil was used at 75 μM as a negative control.

For the sphere-forming assay, the cells were seeded into a 96-well Ultra-Low Attachment Surface Culture Plate (Corning) at 1, 10, 100, and 1000 cells/wells in DMEM/F12 medium supplemented with 20 ng/mL basic fibroblast growth factor (R&D Systems, Minneapolis, MN, USA) and 20 ng/mL epidermal growth factor (R&D Systems) for 7 days. The sphere-positive wells were counted, and CSC frequency and statistical analysis were performed at the ELDA web site (http://bioinf.wehi.edu.au/software/elda) [20].

Xenograft transplantation experiments using animals were performed in accordance with the institutional guidelines for the use of laboratory animals. PANC-1 clone #2 and clone #4 cells were suspended at 1000 or 10000 cells in 100 μl PBS mixed with Matrigel (BD) at a 1:1 volume and injected subcutaneously into the backs of 4-6-week-old female BALB/c-nu/nu mice. Tumor size was assessed weekly using a caliper and calculated using following formula: tumor size (mm^3^) = (longest diameter × shortest diameter^2^)/2.

### Statistical analysis

Student’s *t*-test was used to compare two groups. P<0.05 was considered significant difference. Correlation between expressions of *ABCG2* and %MFI increase was analyzed by Pearson’s correlation coefficient.

## Results

### The pancreatic cancer cell PANC-1 shows heterogeneous 5-ALA staining

**Fig 1:**
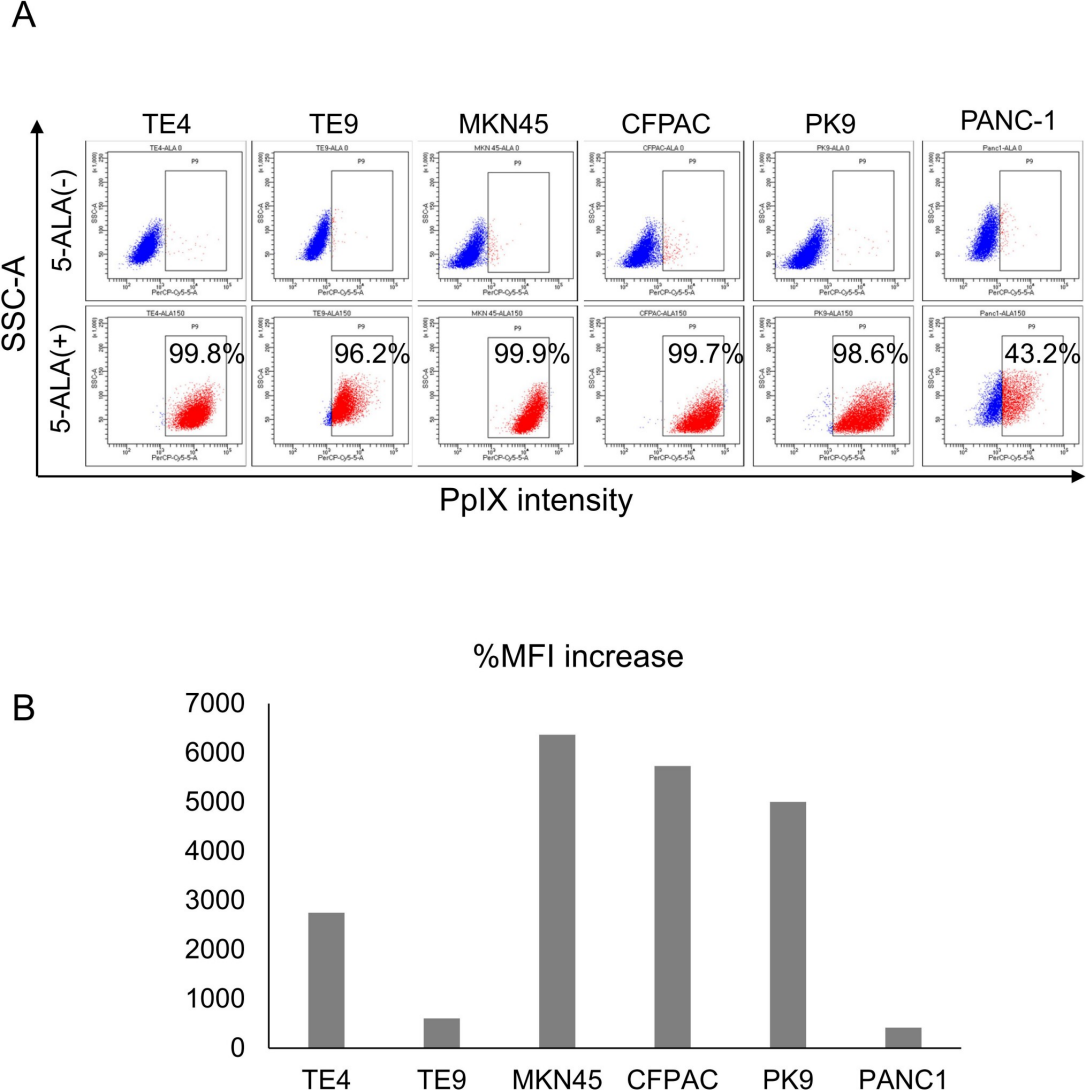
5-ALA staining of gastrointestinal cancer cells. **(A) FACS analysis of 5-ALA-stained gastrointestinal cancer cells.** Esophageal cancer cells (TE4 and TE9), gastric cancer cells (MKN45), and pancreatic cancer cells (CFPAC, PANC-1, and PK9) were stained with 5-ALA (150 μg/mL) for 4 h and analyzed by flow cytometry. Non-stained cells were used as negative controls. Percentages indicate the positive rates of PpIX intensity. **(B) Summary of percent mean fluorescent increase (%MFI)**.

PDD based on 5-ALA-induced PpIX has been applied to several malignancies based on safety and specificity; however, the molecular aspects that define the efficacy of PDD are unknown. In this study, we analyzed several gastrointestinal cancer cell lines to investigate differences in PpIX accumulation. Although most of the TE4, TE9, MKN45, CFPAC, and PK9 cells showed high PpIX intensity with a positive rate of almost 100%, PANC-1 cells showed lower PpIX intensity with a positive rate of 43.2% (Fig 1A). To analyze in detail, %MFI increase was calculated, and PANC-1 cells showed also the lowest %MFI increase (Fig 1B).

### Single cell analysis reveals clonal variation for 5-ALA staining

**Fig 2:**
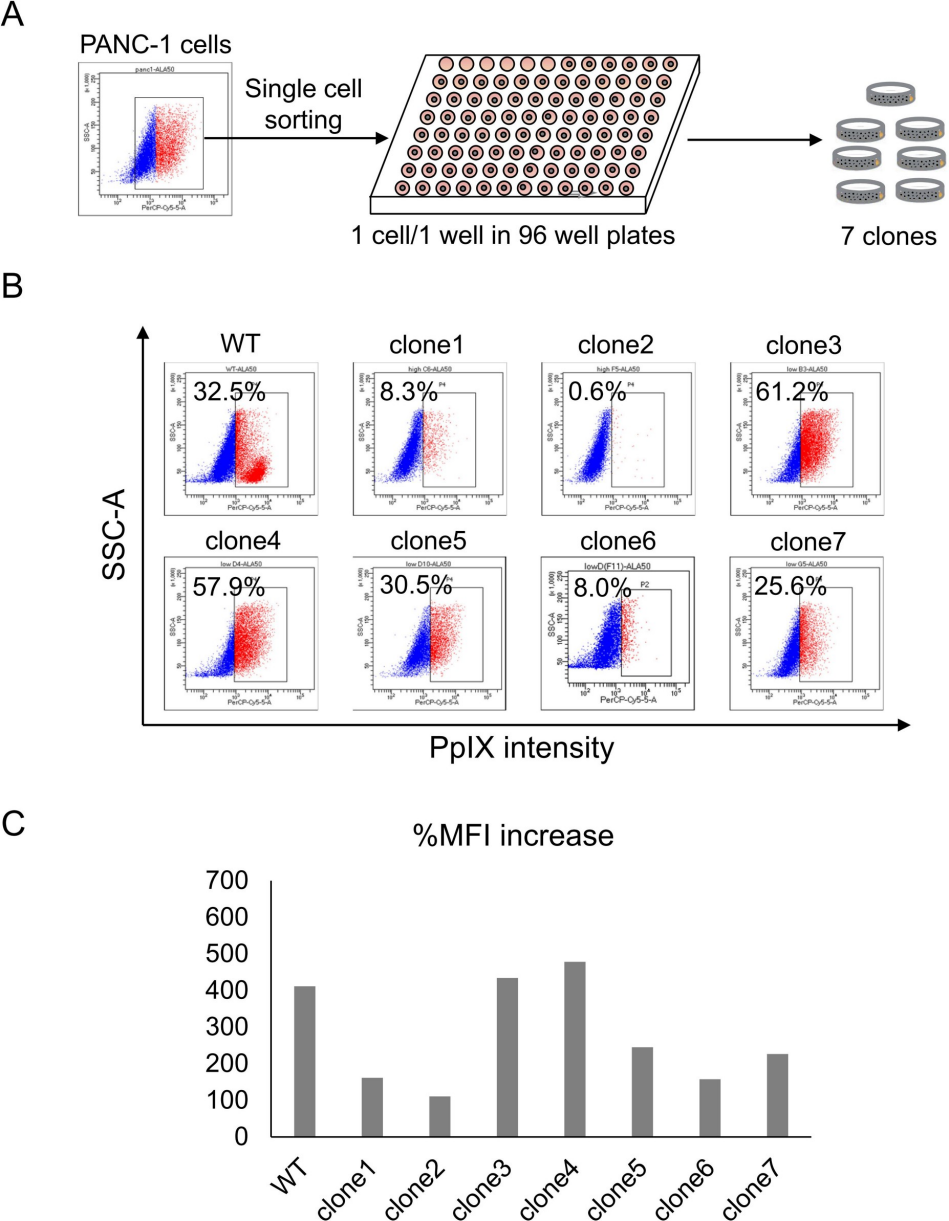
Establishment of PANC-1 clone cells. **(A) Schematic summary of single cell sorting of PANC-1 cells. (B) FACS analysis of 5-ALA-stained PANC-1 clone cells.** Non-stained cells were used as negative controls. Percentages indicate the positive rates of PpIX intensity. **(C) Summary of percent mean fluorescent increase (%MFI)**.

Since PANC-1 cells showed heterogeneous 5-ALA staining pattern, we established PANC-1 clone cells to analyze the heterogeneity of PpIX accumulation at the single cell level (Fig 2A). PANC-1 clone cells were stained with 5-ALA and the accumulation of PpIX was analyzed flow cytometry. The results revealed a heterogeneous PpIX accumulation pattern between the PANC-1 clone cells (Fig 2B). Clone #2 cells showed only a 0.6% positive rate, whereas clones #3 and #4 showed a greater than 50% positive rate, which was higher than that observed in W/T PANC-1 cells. These data indicate that 5-ALA staining was heterogeneous, and it was difficult to detect PpIX accumulation in some PANC-1 clone cells. Clone #2 cells showed the lowest %MFI increase as well (Fig 2C).

### Gene screening reveals that ABCG2 is a candidate for low-5-ALA staining

**Fig 3:**
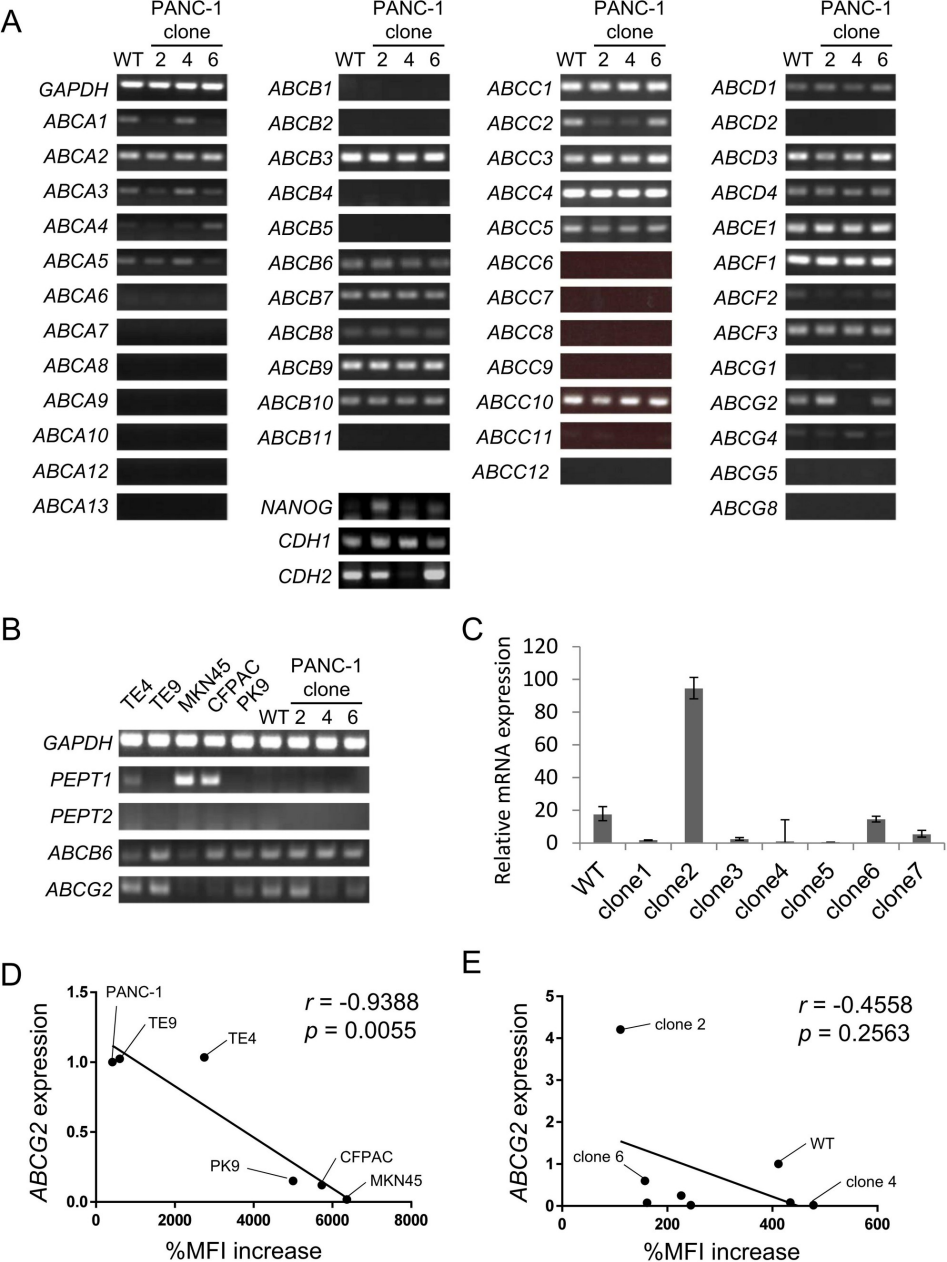
Expression of ABC transporter genes. **(A) RT-PCR analysis of PANC-1 clone cells.** The expression of ABC transporter genes, *NANOG*, *CDH1* and *CDH2* in PANC-1 W/T cells, clone #2 cells, clone #4 cells, and clone #6 cells was analyzed by RT-PCR. GAPDH was used as an internal positive control. **(B) RT-PCR analysis of gastrointestinal cancer cells.** *PEPT1*, *PEPT2*, *ABCB6* and *ABCG2* expression was analyzed by RT-PCR. GAPDH was used as an internal positive control. **(C) qRT-PCR analysis of *ABCG2* expression.** *ABCG2* expression was analyzed by qRT-PCR. Data are shown as the mean ± standard deviation. **(D) Correlation of expressions of *ABCG2* and %MFI increase.** Correlation of expression of quantitative ABCG2 expressions of gastrointestinal cancer cell lines and %MFI increase was analyzed by Pearson’s correlation coefficient. **(E) Correlation of expressions of *ABCG2* and %MFI increase.** Correlation of expression of quantitative ABCG2 expressions of PANC-1 clone cells and %MFI increase was analyzed by Pearson’s correlation coefficient.

To elucidate the mechanism underlying the heterogeneous 5-ALA staining, we focused on transporters that are responsible for 5-ALA uptake and PpIX efflux. Previous studies revealed that oligopeptide transporters PEPT1 and PEPT2 are responsible for 5-ALA uptake [21–23]. Thus, we analyzed the expression of *PEPT1* and *PEPT2* by RT-PCR; however, there was no significant difference in their expression between the PANC-1 clone cells (Fig 3B). We then analyzed ABC transporters using ABC transporter gene-specific primers, because ABC transporters are related to 5-ALA uptake and PpIX efflux (Fig 3A). ABCB6 is responsible for 5-ALA uptake, while ABCG2 is responsible for PpIX efflux [24, 25]. However, the expression of *ABCB6* was equivalent between W/T PANC-1 cells and clones #2, #4, and #6. *ABCG2* expression was high in PANC-1 clone #2 cells and low in clone #4 cells. No other ABC transporter genes showed a significant difference in their expression between the PANC-1 clone cells. Therefore, we focused on the *ABCG2* gene. Genes related to accumulation of PpIX including *PEPT1*, *PEPT2*, *ABCB6* and *ABCG2* expression were analyzed in other cell lines (Fig 3B). PANC-1, TE4 and TE9 cells expressed ABCG2, but MKN45 and CFPAC cells did not. MKN45 and CFPAC cells express PEPT1 at high levels, but other cell lines barely expressed *PEPT1* and *PEPT2*. To confirm the expression of *ABCG2*, we performed qRT-PCR and found that PANC-1 clone #2 cells showed the highest level of *ABCG2* expression, whereas clones #4 and #5 showed the lowest expression levels (Fig 3C). These data indicate an inverse correlation between the expression level of the *ABCG2* gene and PpIX-positive rates.

To analyze the relation of quantitative *ABCG2* expression and %MFI increase correlation coefficient (CI) was calculated. The expressions of *ABCG2* in cancer cell lines and %MFI increase showed statistically significant inverse correlation (r = -0.9388, p = 0.0055) (Fig 3D). The expressions of *ABCG2* in PANC-1 clone cells and %MFI increase showed tendency to inverse correlation but did not reach statistical significance (r = -0.4558, p = 0.2563) (Fig 3E). All these data suggest the inverse relation of *ABCG2* expression and PpIX intensity.

### ABCG2 knockdown using siRNAs reveals that ABCG2 has a role in low 5-ALA staining

**Fig 4:**
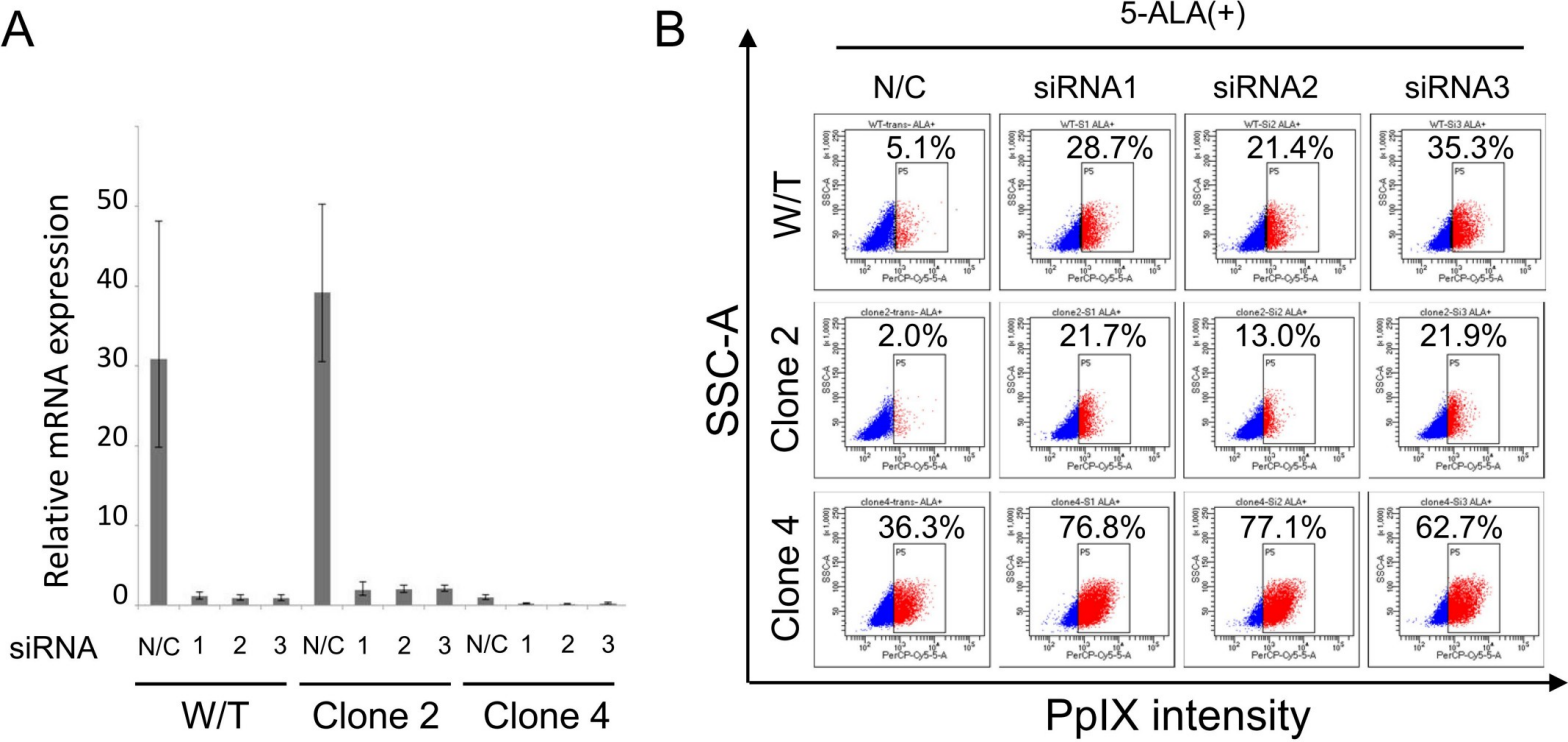
Knockdown of ABCG2 in PANC-1 clone cells increases 5-ALA staining. **(A) qRT-PCR analysis of *ABCG2* expression.** PANC-1 W/T cells, clone #2 cells, and clone #4 cells were transfected with ABCG2 siRNAs. At 2 days after transfection, *ABCG2* expression was analyzed by qRT-PCR. Data are shown as the mean ± standard deviation. **(B) FACS analysis of 5-ALA-stained ABCG2 knockdown cells.** PANC-1 W/T cells, clone #2 cells, and clone #4 cells were transfected with ABCG2 siRNAs. At 2 days after transfection, the cells were stained with 5-ALA (150 μg/mL) for 4 h and analyzed by flow cytometry. Percentages indicate the positive rates of PpIX intensity.

A previous study revealed that ABCG2 has a role in PpIX efflux [24]; thus, we analyzed the role of ABCG2 in 5-ALA staining using siRNAs. The *ABCG2* gene was knocked down using *ABCG2*-specific siRNAs in PANC-1 W/T, clone #2 cells, and clone #4 cells, and knockdown efficiency was confirmed by qRT-PCR (Fig 4A). Knockdown of *ABCG2* increased PpIX-positive rates in W/T cells, clone #2 cells, and clone #4 cells (Fig 4B), indicating that ABCG2 has a role in low 5-ALA staining rates.

### ABCG2-high cells are enriched with CSCs, but ABCG2 is not related to cancer stemness

**Fig 5:**
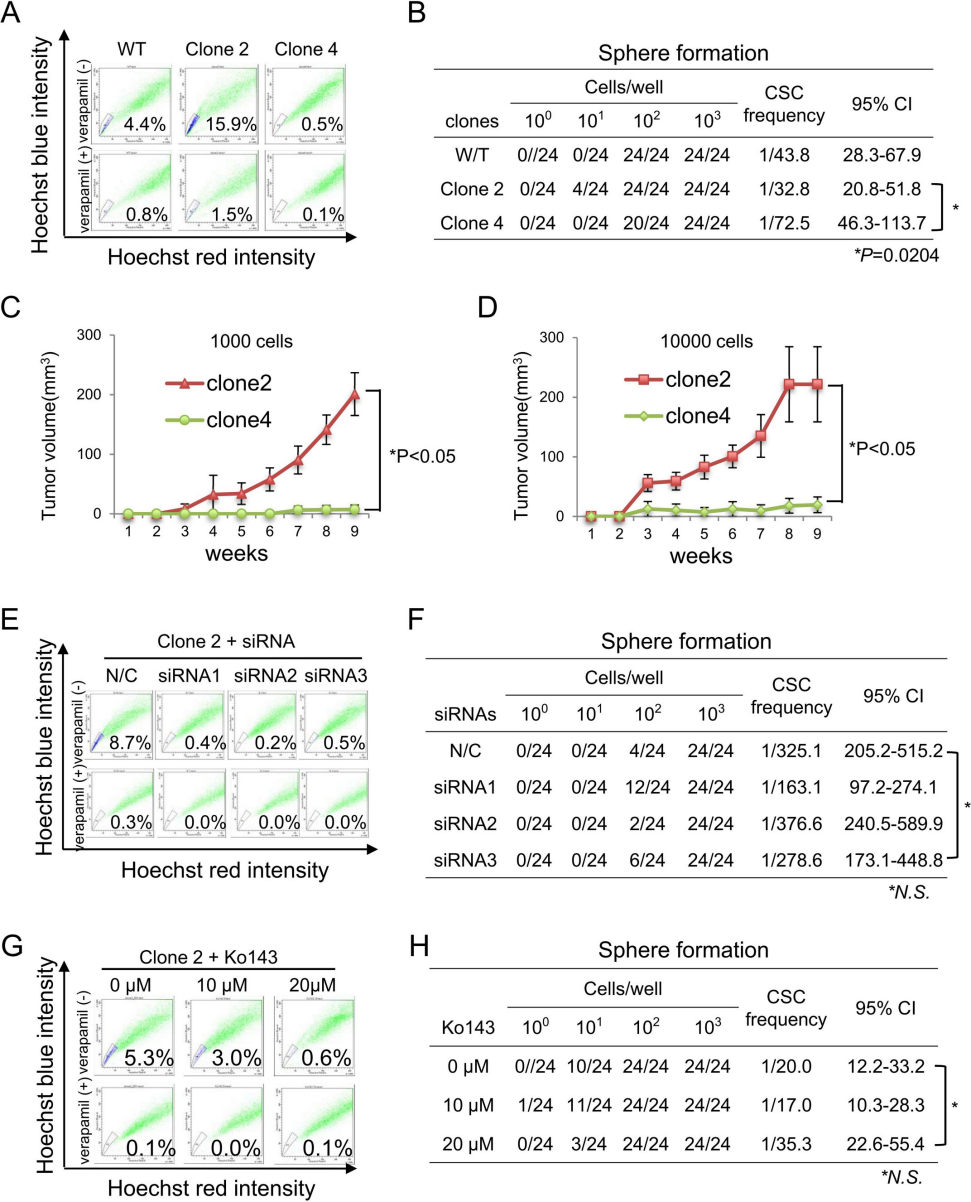
Inhibition of ABCG2 decreases SP cells, but not sphere-forming ability. **(A) SP analysis of PANC-1 cells.** PANC-1 W/T cells, clone #2 cells, and clone #4 cells were stained with Hoechst 33342 and analyzed by flow cytometry. Verapamil was used as a negative control. Percentages indicate the rates of SP cells. **(B) Sphere-forming ability of PANC-1 cells.** PANC-1 W/T cells, clone #2 cells, and clone #4 cells were seeded in 96-well ultra-low attachment plates at 1, 10, 100, and 1000 cells/well. At 1 week later, sphere-forming wells were counted, and CSC frequency was calculated using the ELDA website. The chi-square test was performed to determine statistically significant differences. **(C and D) Tumorigenicity of PANC-1 clone #2 and #4 cells.** Growth curves of tumors derived from PANC-1 clone #2 and clone #4 cells. 1000 cells (C) and 10000 cells (D) were injected into BALB/c-nu/nu mice, and tumors were measured weekly. Data are shown as means±SD. Asterisks indicates a statistically difference (P<0.05). **(E) SP analysis of ABCG2 knockdown PANC-1 clone #2 cells.** PANC-1 clone #2 cells were transfected with ABCG2 siRNAs. At 2 days after transfection, the cells were analyzed by an SP assay. Verapamil was used as a negative control. Percentages indicate the rates of SP cells. **(F) Sphere-forming ability of ABCG2 knockdown PANC-1 clone #2 cells.** PANC-1 clone #2 cells were transfected with ABCG2 siRNAs. At 1 day after transfection, the cells were seeded in 96-well ultra-low attachment plates at 1, 10, 100, and 1000 cells/well. At 1 week later, sphere-forming wells were counted, and CSC frequency was calculated using the ELDA website. The chi-square test was performed to determine statistically significant differences. N.S.: No significant difference. **(G) SP analysis of Ko143-treated PANC-1 clone #2 cells.** PANC-1 clone #2 cells were treated with Ko143 at a concentration of 0, 10, or 20 μM for 24 h and analyzed by an SP assay. Verapamil was used as a negative control. Percentages indicate the rates of SP cells. **(H) Sphere-forming ability of Ko143-treated PANC-1 clone #2 cells.** PANC-1 clone #2 cells were seeded in 96-well ultra-low attachment plates at 1, 10, 100, and 1000 cells/well with Ko143 at a concentration of 0, 10, or 20 μM. At 1 week later, sphere-forming wells were counted, and CSC frequency was calculated using the ELDA website. The chi-square test was performed to determine statistically significant differences. N.S.: No significant difference.

Several studies have revealed that a highly tumorigenic subpopulation, termed CSCs, are enriched in SP cells, and SP cells are defined by ABCG2 expression [18, 26–29]. Therefore, we hypothesized that PANC-1 clone #2 cells are enriched with CSCs and performed an SP assay and sphere formation assay. PANC-1 clone #2 cells showed an SP rate of 15.9%, whereas W/T cells and clone #4 cells showed SP rates of 4.4% and 0.5%, respectively (Fig 5A). A sphere formation assay revealed that PANC-1 clone #2 cells has higher sphere formation ability than clone #4 cells (P = 0.0204) (Fig 5B). To analyze the expressions of stem cell genes in PANC-1 clone cells, we performed RT-PCR. Clone #2 cells showed relative high expression of *NANOG*, but clone #4 cells did not (Fig 3A). Clone #2 cells showed higher *CDH2* gene, an epithelial-mesenchymal transition marker than that in clone #4 cells suggesting that clone #2 cells are enriched with CSCs and have EMT-like phenotype (Fig 3A). Furthermore, clone #2 cells showed higher tumorigenicity than clone #4 cells (Fig 5C and 5D), indicating that clone #2 cells contain a higher proportion of CSCs compared with W/T or clone #4 cells. Since PANC-1 clone #2 cells showed the highest level of ABCG2 expression, we analyzed the relationship between ABCG2 expression and CSC phenotype. Knockdown of *ABCG2* using siRNAs and inhibition of ABCG2 using Ko143 decreased the SP rate (Fig 5E and 5F), whereas knockdown of *ABCG2* and inhibition of ABCG2 did not decrease sphere formation (Fig 5G and 5H), indicating that ABCG2 expression is not related to tumor formation.

## Discussion

5-ALA PDD/PDT have been approved for dermatologic malignancies and gliomas and have been applied to several malignant tumors [30]. A phase III clinical trial of surgery with 5-ALA PDD for malignant gliomas revealed that it significantly improved progression-free survival [2], indicating that 5-ALA PDD is an effective approach for the diagnosis of malignant tumors. However, most of the patients enrolled in that study showed disease progression, demonstrating that detection efficacy needs to be improved. In this regard, several analyses at the molecular level have been reported.

ABCG2 (breast cancer resistance protein, BCRP) transports various endogenous and exogenous chemicals [31], thereby mediating drug resistance and affecting the pharmacological action of many compounds in different drug-resistant cancer cells [32–34]. A study using diffuse large B cell lymphoma cells revealed that ABCG2 is a transcriptional target of the hedgehog signaling transcription factor GLI1 [35]. Sonic hedgehog (SHH) signaling is one of the major signaling pathway activated in pancreatic cancer [36]. A phase Ib/II clinical trial using Vismodegib, an SHH inhibitor, plus gemcitabine did not improve progression-free or overall survival in patients with metastatic pancreatic cancer [37], suggesting difficulties in targeting SHH signaling. Regarding hedgehog signaling, a study using PANC-1 cells revealed that sphere-forming CSCs showed high expression of the epithelial-mesenchymal transition transcription factor SNAI1 and hedgehog signaling ligand SMO [38], indicating that the activation of SHH signaling, CSC phenotype, and epithelial-mesenchymal transition are deeply related to each other. In the present study, ABCG2-high PANC-1 clone #2 cells showed high sphere-forming ability compared with ABCG2-low clone #4 cells. Therefore, ABCG2-high pancreatic CSCs might be well targeted by 5-ALA PDT using an ABCG2 inhibitor, and this approach could be another way to target SHH signaling-activated pancreatic cancer cells. ABCG2 knockdown using siRNAs increased accumulation of PpIX, however, the PpIX positive rate did not reached 100% even in clone #4 cells. PANC-1 cells express both ABCB6 and ABCG2, however the expression levels of PEPT1 and PEPT2 were very low (Fig 3B). Therefore, the accumulation rates of PpIX in PANC-1 clone cells might also depend on also low expressions of PEPT1 and PEPT2. A recent study revealed that C6 rat glioma cells with low PpIX accumulation are enriched with glioma stem cells [39]. Interestingly, the accumulation of PpIX in glioma stem cells was not enhanced by ABCG2 inhibition using reserpine, but was enhanced by deferoxamine-mediated iron chelation, suggesting that PpIX accumulation depends on several factors.

ABCB6 is a transporter for the uptake of 5-ALA, and high ABCB6 expression is related to high PpIX accumulation [25]. High ABCB6 expression is related to the response to neoadjuvant chemotherapy in breast cancer and the progression of prostate cancer [40, 41]. Although, the molecular mechanisms by which ABCB6 expression is related to chemotherapy resistance or cancer progression are unknown, ABCB6-high malignant cancers are considered to be suitable targets for 5-ALA PDD/PDT because ABCB6-high cancer cells are thought to accumulate PpIX. However, the expression levels of ABCB6 were equivalent between PANC-1 W/T cells and clone cells by RT-PCR, and ABCB6 expression might not be related to PpIX accumulation in pancreatic cancer cells. These results suggest that ABCB6 expression might not be an accurate marker for predicting the efficacy of 5-ALA PDD/PDT in pancreatic cancer. The accumulation of data using clinical samples from subjects with pancreatic cancer should be conducted in the future.

In summary, we reported that most of the gastrointestinal cancer cell lines examined showed homogenous 5-ALA staining. However, the pancreatic cancer cell line PANC-1 showed heterogeneous 5-ALA staining. High ABCG2 expression might be responsible for low 5-ALA staining, and CSCs are enriched in the ABCG2-high population. Inhibition of ABCG2 improved 5-ALA staining, but had no effect on cancer stemness. These results indicate that ABCG2 expression might become a novel biomarker for poor 5-ALA PDD/PDT efficacy, and the combination of 5-ALA PDD/PDT with an ABCG2 inhibitor might improve efficacy. Finally, CSCs could also be targeted by the combination of 5-ALA PDD/PDT with an ABCG2 inhibitor.

## Supporting information

S1 Table Information of RT-PCR primers. (XLSX)

## Abbreviations

5-ALA: 5-aminolevulinic acid
ABC: ATP-binding cassette
CSC: cancer stem cell
DMEM: Dulbecco’s modified Eagle’s medium
FACS: fluorescence-activated cell sorting
FBS: fetal bovine serum
SP: side population
PBS: phosphate-buffered saline
PDD: photodynamic diagnosis
PDT: photodynamic therapy
PpIX: protoporphyrin IX
qRT-PCR: quantitative RT-PCR
siRNA: small interfering RNA
W/T: wild-type
